# Congenital myopathy is caused by mutation of *HACD1*

**DOI:** 10.1093/hmg/ddt380

**Published:** 2013-08-09

**Authors:** Emad Muhammad, Orit Reish, Yusuke Ohno, Todd Scheetz, Adam DeLuca, Charles Searby, Miriam Regev, Lilach Benyamini, Yakov Fellig, Akio Kihara, Val C. Sheffield, Ruti Parvari

**Affiliations:** 1 Shraga Segal Department of Microbiology, Immunology and Genetics, Faculty of Health Sciences and; 2 National Institute of Biotechnology in the Negev, Ben Gurion University of the Negev, Beer Sheva 84105, Israel,; 3 Genetic Institute, Assaf Harofeh Medical Center, Zerifin 70300, Israel,; 4 The Sackler School of Medicine, Tel Aviv University, Tel Aviv 69978, Israel,; 5 Laboratory of Biochemistry, Faculty of Pharmaceutical Sciences, Hokkaido University, Kita 12-jo, Nishi 6-Chome, Kita-ku, Sapporo 060-0812, Japan,; 6 Department of Ophthalmology,; 7 Department of Biomedical Engineering and; 8 Department of Pediatrics, Division of Medical Genetics, Howard Hughes Medical Institute, University of Iowa, Iowa City, IA 52242, USA and; 9 Department of Pathology, Hadassah–Hebrew University Medical Center, Jerusalem 91120, Israel

## Abstract

Congenital myopathies are heterogeneous inherited diseases of muscle characterized by a range of distinctive histologic abnormalities. We have studied a consanguineous family with congenital myopathy. Genome-wide linkage analysis and whole-exome sequencing identified a homozygous non-sense mutation in *3-hydroxyacyl-CoA dehydratase 1 (HACD1*) in affected individuals. The mutation results in non-sense mediated decay of the *HACD1* mRNA to 31% of control levels in patient muscle and completely abrogates the enzymatic activity of dehydration of 3-hydroxyacyl-CoA, the third step in the elongation of very long-chain fatty acids (VLCFAs). We describe clinical findings correlated with a deleterious mutation in a gene not previously known to be associated with congenital myopathy in humans. We suggest that the mutation in the *HACD1* gene causes a reduction in the synthesis of VLCFAs, which are components of membrane lipids and participants in physiological processes, leading to congenital myopathy. These data indicate that *HACD1* is necessary for muscle function.

## INTRODUCTION

Congenital myopathies are a distinct group of genetically heterogeneous inherited diseases of muscle that manifest clinically in early life, infancy or variants with later onset, and are characterized by a range of distinctive abnormalities upon muscle biopsy. The common forms of congenital myopathies can be subdivided based on the predominant pathologic features observed under light and electron microscopy into the following categories: (i) myopathies with protein accumulations (including nemaline or nemaline rod myopathies); (ii) myopathies with central cores (regions devoid of oxidative activity); (iii) centronuclear myopathies (CNMs) with abnormally localized nuclei, usually centrally placed and (iv) myopathies with congenital fiber type disproportion (CFTD) characterized by selective hypotrophy or atrophy of type 1 (slow twitch) fibers, with no other structural changes. The precise histologic diagnosis of congenital myopathies is sometimes difficult to make due to overlapping features. In addition, the clinical and histologic abnormalities can evolve with time, and diagnosis may be deferred until the distinct phenotype is apparent. Abnormal excitation–contraction coupling may be a common theme in the congenital myopathies, either as a result of malformed contractile filaments in the case of the nemaline myopathies or disruption of calcium homeostasis at the level of the triad (the smallest functional component of the myofiber that includes the T-tubule and sarcoplasmic reticulum) in the case of the centronuclear/myotubular and core myopathies (1). Congenital myopathies can be caused by mutations in different genes, and many of the causative genes are associated with >1 histologic diagnosis (2). The number of genes associated with congenital myopathies is now reportedly >20, and it is clear that additional genes are yet to be identified.

Because of the clinical and genetic heterogeneity of congenital myopathies, molecular diagnosis is of paramount importance for the clinical assessment and has implications for treatment.

**Figure 1.**
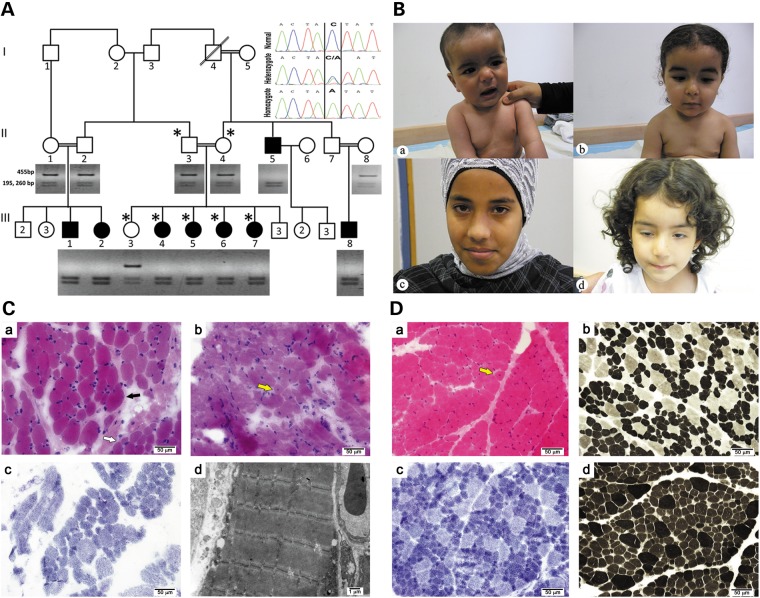
The myopathy family: genetics, clinical histolopathological and EM findings. (**A**). Segregation of the *HACD1* mutation in the pedigree. Digestion of the 455 bp amplicon of exon 6 with SspI containing the sequence variation NM_014241:c.744C > A results in cleavage into 195 and 260 bp fragments. Inset: sequence of the corresponding c.744C > A mutation resulting in p.Tyr248Stop. Patients were homozygous for the mutation; parents and the healthy sibling were heterozygous, and a healthy control is homozygous for the normal sequence. The genotyped individuals are marked by ‘*’. (**B**) Photographs of patients: (a) Patient III-1 at the age of 8 months sitting with support. Note facial weakness and dropping shoulders. (b) Patient III-2 at age 1 and 8 months. Note facial weakness, drooping shoulders and pectus excavatus. (c) and (d) correlate to Patients III-5 and III-6, ages 14 and 3 years, respectively. Note facial weakness and ptosis of the right eye in the latter. Permissions from guardians were granted for all shown photographs. (**C**) Histology and EM of core needle biopsy: (a)Frozen sections from Patient III-8 at the age of 2 years stained with H&E display focal variation in myofiber diameter (black arrow). Large, hypertrophic myofibers (most of them 35–40 μm in diameter) are scattered among smaller myofibers (normal diameter for age (∼20 μm) and small for age (∼13–16 μm in diameter)), occasionaly in small groups (white arrow). (b) Only isolated internally (centrally) displaced nuclei are seen (yellow arrow). (c) On NADH histochemical stain most hypertrophic myofibers are type 2, while most small fibers are type 1. There are no significant changes in the cytoarchitecture. (d) Electron microscopy is unremarkable. (**D**) Histology of open biopsy: (a) paraffin embedded and frozen sections from Patient III-5 at age 1 year stained with H&E display marked variation in myofiber diameter. In many areas, hypertrophic myofibers (most of them 20–30 μm in diameter) are scattered among smaller myofibers (normal diameter for age ∼18 μm in diameter and small for age ∼10–15 μm in diameter), occasionally in small groups. Only isolated internally (centrally) displaced nuclei are seen (yellow arrow). (b–d) On enzyme-histochemical staining (b, NADH; c, ATPase 4.3; d, ATPase 9.4), most scattered hypertrophic myofibers are type 2, while most (>90%) small fibers are type 1. There is also a relative increase of type 1 myofibers (∼80%).

We describe here the identification of a novel gene that when mutated leads to CFTD myopathy, by using genetic mapping and exome sequencing of a highly inbred family of Bedouin ancestry (Fig. 1A).

## RESULTS

### Clinical and laboratory findings in affected patients

Intermarriage between six first-degree cousins resulted in 13 affected members. Eight affected individuals were clinically evaluated by us, three males and five females (Fig. 1A); the rest had been diagnosed with congenital myopathy by their physicians, but were not available for this study. Age ranged from 6 months to 35 years (mean of 5.9 years). All presented with a severe myopathic phenotype at birth that gradually improved (Table 1). Pregnancies were uneventful in all, without polyhydramnios and with normal fetal movements; however, three had breech presentations requiring cesarian section and one was delivered early due to premature rupture of membranes. All had Apgar scores between 8–9 and 7–10 at 1 and 5 min, respectively. Birth weight, head circumference and length were appropriate for gestational age in all patients. Severe hypotonicity was detected in all during the neonatal period with head lag, absent deep tendon reflexes and reduced newborn reflexes. No patients had fasciculations. Pectus excavatum was detected in two patients and frog position in two of eight patients. A weak cry was detected in all patients and recurrent apnea leading to prolonged newborn hospitalization occurred in three patients. Severe facial weakness was noted in all with drooling and reduced sucking reflexes in five out of eight patients, lasting from 1 week up to 2.5 years of age. One patient required a gastrostomy and recurrent aspiration pneumonia occurred in two patients. A marked delay in achieving motor milestones was detected in all: head raise could be recorded at the age of 3–4 months in one patient, rolling was detected between 6 months and 2 years of age, sitting ranged from 6 months in one patient to 2.5 years in two cases (mean age 1 year); standing with support ranged from 1.5 to 2.5 years of age (mean 2 years). Walking with support was noted between 1.5 and 2.5 years; two patients gained steady walk at 2.5 years but in one patient even at the age of 11.5 years a waddling gate was noted. Recurrent stumbling was noted in the 12- and 14-year-old patients. Climbing steps required assistance at 2.5 years of age and lasted up to the age of 12 years in one patient. Gower sign was positive in all and sustained until 14 years in one patient. All patients had reduced muscle tone, which was most notable proximally. Except for delayed motor skills, cognition was within normal limits in all. The oldest patient, age 35 years, works as a truck driver with daily heavy lifting without any limitations. He neither had facial weakness nor Gower sign, but has reduced deep tendon reflexes with bilateral pes cavus that seem to be a remnant of his childhood severe myopathy. Otherwise his neurologic examination was within the normal range. Minor cardiac defects such as PDA, ASD and PFO were detected in three out of eight patients and resolved with time. These were unlikely to be related to the congenital myopathy defect because these findings were also detected in three out of nine otherwise healthy siblings.

**Table 1.** Major clinical features in affected individuals with p.Tyr248Stop mutation in HACD1

Clinical feature	Number of patients with the signs/number of patients examined
Age 0–1.5 years (all patients)	
Normal pregnancy	7/8
Breech presentation and Cesarean Section	3/7
Normal APGAR scores	7/7
Respiratory fetal distress syndrome	1/7^a^
Severe hypotonicity	8/8
Absent deep tendon reflexes	8/8
Weak cry	7/7
Recurrent apnea of newborn	3/7^b^
Feeding problems	5/7
Recurrent aspirations	2/7
Cardiac findings	
Resolved PDA/PFO/ASD	3/8
Age 2.5—14 years (Patients II-5, III-4–7)	
Facial weakness	4/5
Decreased muscle tone	5/5
Absent deep tendon reflexes	5/5
Delayed motor milestones	5/5
Waddling gate	5/5
Gower sign positive	5/5
Age 35 years (Patient II-5)	
Absent deep tendon reflexes	1/1
Normal gait, posture and muscle tone	1/1
Residual severe pes cavus	1/1

^a^Preterm 33 weeks of gestation required a few days of mechanical ventilation.

^b^Requiring longer admission to Neonatal Intensive Care Unit.

PDA, patent ductus arteriosus; PFO, patent foramen ovale; ASD, atrial septal defect.

Laboratory evaluation in affected individuals consisted of serum tests including CPK, liver enzymes, amino acids, lactate, pyruvate, total and free carnitine, ammonia, urine organic acids and beta oxidation activity in lymphocytes all of which were normal. Saturated very long-chain fatty acids (VLCFAs) levels were determined for Patient III-4 at the age of 1 year by GC–MS analysis of methylesters and found to be normal: ratios: C26/C22 0.015 (range = 0.018 ± 0.009), C24/C22 0.60 (range = 0.68 ± 0.15) and C22 26.4 µg/ml (range = 10–35). The pristanic and phytanic levels were 0.05 µg/ml (range = 0.01–0.4) and 1.5 µg/ml (range = 0.5–3.5), respectively. Brain ultrasounds were performed at the age of 2–6 months in two patients with normal results. Electrocardiograms were repeatedly shown to be normal in all examined patients. Electromyography (EMG) was performed in two patients: in one (Patient II-2) the results were non-conclusive at the age of 2 months, while in the second (Patient II-1) needle EMG performed at the age of 1 year was compatible with a myopathic lesion; no denervation activity was detected and numerous low-voltage polyphasic motor units, most evident in quadriceps, were recorded. Nerve conduction studies were performed on one patient and proved to be normal. Electron microscopy of a skin biopsy in an attempt to evaluate the patient for neuroaxonal dystrophy was normal. Figures demonstrating myopathy and pectus excavatum are presented in Fig. 1B. Muscle biopsies (Fig. 1C and D) were performed in two patients. Patient III-8 had a core needle biopsy (Fig. 1C), at the age of 2 years (site not recorded), had shown mild myopathic changes, mainly type 1 fiber smallness. However, the biopsy was very small and the changes were not conspicuous enough for definitive diagnosis. Only rare myofibers (<1% of the entire sample) displayed internally displaced nuclei. Patient III-5 had a right quadriceps open biopsy (Fig. 1D) at the age of 1 year. The main finding was decreased size of type 1 myofibers. About 100 myofibers were measured and the range in diameter of most type 1 fibers was 10–15 μm (normal diameter for age is ∼18 μm). Also, a relative increase in the number of type 1 myofibers (∼80% of all myofibers) was noted. Scattered very small (5–8 μm in diameter) type 1 myofibers were noted. In addition, scattered hypertrophic type 2 myofibers (20–30 μm in diameter) were noted. Only rare myofibers (<1% of the entire sample) displayed internally displaced nuclei. Nevertheless, due to the patient's young age this finding may have significant implications. In both biopsies, there were no signs of necrosis, regeneration or any other specific structural change in the myofibers. Neither cores, minicores nor rods were identified. Neither significant endomysial fibrosis nor inflammatory infiltrates were seen. The blood vessels were unremarkable. In the modified Gomori stain, no additional changes were noted. On enzyme-histochemical studies (ORO; PAS; PAS + D; ATPase 9.4, 4.3; NADH; SDH; COX), there were no significant changes in the cytoarchitecture. COX staining was normal. There was no evidence of glycogen or lipid excess in the myofibers. Additional immunohistochemical stains were performed on the biopsy of Patient III-5 including immunohistochemical stains for spectrin, dystrophin (dys1, dys2, dys3), utrophin, sarcoglycans (alpha, beta, gamma, delta), laminin beta-1, merosin (300 kDa, 80 Da), dysferlin, caveolin-3, perlecan, collagen IV, collagen VI and emerin (Supplementary Material, Fig. S1). These were all normal. Electron microscopy performed on the biopsy of Patient III-8 did not reveal any diagnostic abnormalities (Fig. 1Cd, Supplementary Material, Fig. S2). Respiratory chain activity was normal.

In the absence of any specific structural changes (including significant increase of central nuclei, rods, cores, multi/mini-cores or obvious dystrophic type changes) the main differential diagnosis of the findings includes congenital myopathy with features of CFTD and the possibility of biopsy at too young of an age to visualize other specific pathology such as CNM. A severe onset at birth with gradual improvement and central nuclei in the muscle biopsy can be seen in congenital myotonic dystrophy (3). However, the autosomal recessive mode of inheritance in this family and the lack of a *DMPK* trinucleotide repeat mutation exclude this possibility. Although mutations in other genes have been described to be associated with CFTD (ACTA1, SEPN1, TPM3, RYR1, MYH7) (2), all were excluded because the patients did not show homozygosity at any of these loci (see below).

### Homozygosity mapping and identification of the mutation in *HACD1*

In order to pursue a molecular diagnosis in this family, we performed genotyping on four patients (III-4, III-5, III-6 and III-7), their parents and a healthy sibling (III-3). Based on the consanguinity in the family we hypothesized homozygosity by descent of a recessive mutation as the likely cause of the disorder. Therefore, we searched for homozygous regions consistent with linkage. Three homozygous blocks shared by the three affected individuals, heterozygous in the parents, and not homozygous in the unaffected sib were identified. These candidate regions (each with a conservative lod score of ∼1.8 at theta = 0) were each >4 Mb in length and the combined length of the three regions was 38 Mb. Because the regions of linkage were suggestive, but not statistically significant, we reasoned that an appropriate next step in identifying the disease gene was exome sequencing. We hypothesized that the regions of homozygosity (on chromosomes 6, 10 and 14) could be evaluated by exome sequencing to identify the most likely disease causing mutation. Whole-exome sequencing was performed on Patient III-4 using NimbleGen v2 exome capture. Analysis of the variations in the homozygous regions revealed three novel variations: missense changes in *AKD1* and *MRC1L1* and a non-sense change in *3-hydroxyacyl-CoA dehydratase 1* (*HACD1*, also known as *protein tyrosine phosphatase-like member A*, *PTPLA*).

**Figure 2.**
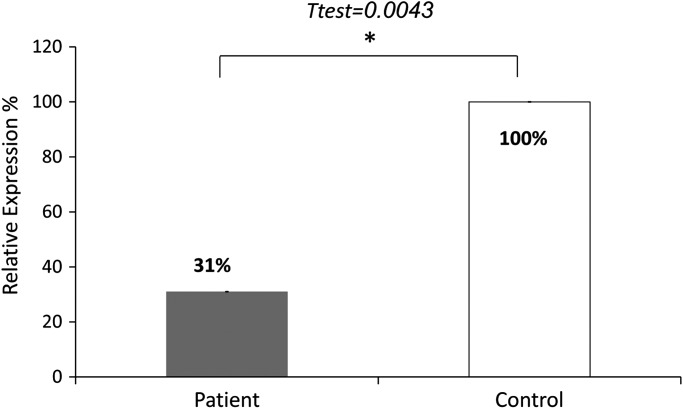
Quantitative PCR of *HACD1* cDNA in a frozen sample biopsy of a patient compared to controls. The results represent three experiments each done in duplicates. The qPCR data was analyzed with the ABI 7500 Software V2.0.3 (Δ*C*_t_ method; normalization against GAPDH). Values are expressed as relative expression to the control muscle, means ± s.e.m. (not visible). Difference between groups was determined by the two-tailed Student's *t*-test (*P* = 0.0043). The *HACD1* primers yielded a linear standard curve with an *R*^2^ = 0.99.

Since a SINE exonic insertion in *HACD1* that leads to multiple splicing defects was reported to segregate with an autosomal recessive CNM in dogs (4), we verified whether the non-sense change in *HACD1* could contribute to the disease in the family. The sequence variation NM_014241:c.744C > A; p.Tyr248Stop, in exon 6, was verified by Sanger sequencing in family members III-3–7 and their Parents II-3 and II-4. All patients were homozygous for the non-sense mutation and the healthy sibling and the parents were heterozygous as tested by analysis of an SspI restriction site created by the variation (Fig. 1A inset). This variation was not reported in the online databases dbSNP, 1000 Genomes project and the NHLBI Exome Sequencing Project. The variation was not found in 134 healthy Bedouin controls. The non-sense mutation is expected to cause non-sense mediated degradation of the mRNA since it occurs in the penultimate exon (5). We compared the quantity of HACD1 *mRNA* in the biopsy of Patient III-5 at the age of 1 year to commercially available RNA of control muscle, which revealed a mean reduction of 69% (Fig. 2).

**Figure 3.**
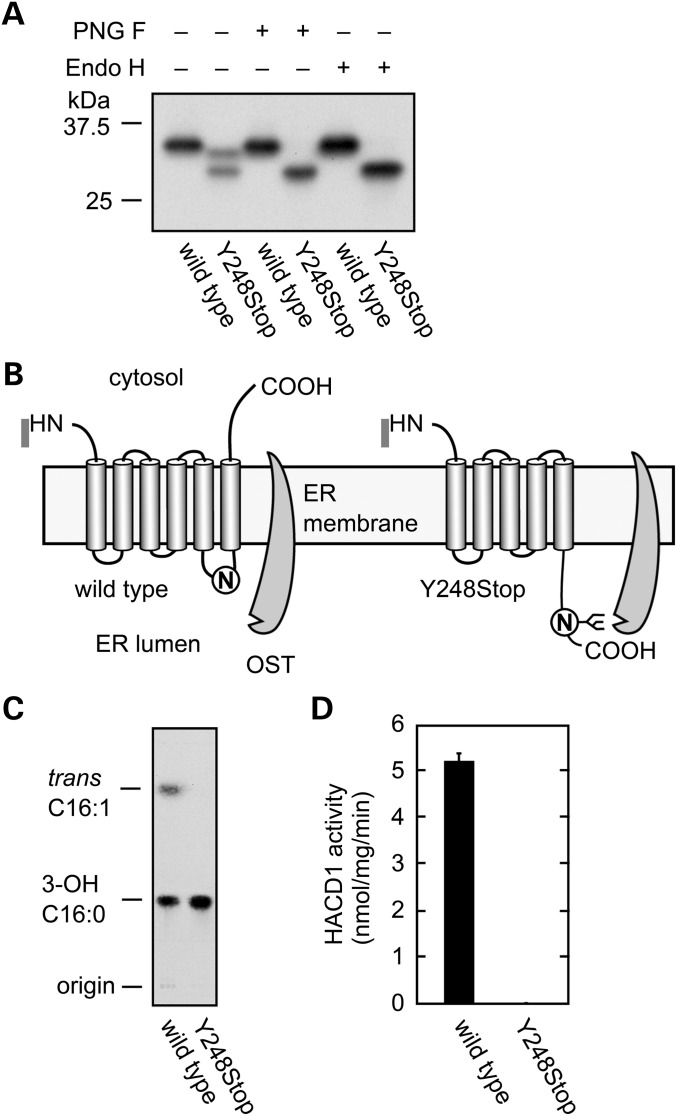
*HACD1* Y248Stop exhibits no activity. (**A**). Production of 3xFLAG-tagged wild-type and mutant (Y248Stop) HACD1 proteins in HEK 293 T following purification using anti-FLAG M2 agarose. Proteins were incubated with buffer, peptide:N-glycosidase F (PNG F), or endoglycosidase H (Endo H) and detected by immunobloting with anti-FLAG antibodies. (**B**). Model for glycosylation of HACD1 (Y248Stop). OST: oligosaccharyltransferase. (**C**). Protein samples were incubated for 10 min at 37°C with [^14^C]3-hydroxypalmitoyl-CoA. After termination of the reactions, lipids were saponified, acidified, extracted and separated by TLC. 3-OH 16:0, 3-hydroxypalmitic acid; *trans* C16:1, 2,3-*trans*-hexadecenoic acid. (**D**). The radioactivity associated with the reaction product 2,3-*trans*-hexadecenoic acid quantified using bioimaging analyzer BAS-2500 (Fuji Photo Film, Tokyo, Japan) and represent the mean (± SD) from three independent experiments.

The 3-hydroxyacyl-CoA dehydratase protein is involved in elongation of VLCFAs (6). This enabled us to perform biochemical analyses of the HACD1 (Y248Stop) mutant protein. HEK 293 T cells were transfected with the wild-type or mutated constructs, and the FLAG-tagged proteins were affinity-purified using anti-FLAG M2 agarose. Although wild-type HACD1 protein was detected as a single band by western blot analysis, 2 protein products were observed for the mutated HACD1 (Y248Stop) (Fig. 3A). We tested the possibility that the slower migrating protein is glycosylated by incubation with Peptide: *N*-glycosidase F (PNG F) or endoglycosidase H (Endo H). Either of these glycosidases eliminated the slower migrating band, indicating that HACD1 (Y248Stop) was glycosylated. The enzymatic activity was verified by incubation of 20 ng proteins with the substrate [^14^C]3-hydroxypalmitoyl-CoA and testing the production of 2,3-*trans*-hexadecenoyl-CoA. The mutation completely abrogated the enzymatic activity (Fig. 3C and D).

## DISCUSSION

Clinical presentations of patients with congenital myopathies are remarkably variable. For example, a few patients with mutations in *MTM1* that had severe hypotonia at birth were still alive at the age of 15 years (7). Also, CNM has been reported in a patient with a homozygous mutation in *RYR1* that improved with age (8,9). Here, we present eight patients of one family with a loss-of-function mutation in *HACD1* with gradual improvement with age: apnea and feeding problems that were detected at infancy had resolved thereafter; waddling gate and Gower sign detected up to age 14 years had resolved in adulthood and the older patient aged 35 years is not affected and functions normally in adulthood, although this patient had hypotonia when young. Interestingly, Labrador Retrievers with canine CNM resulting from a SINE mutation in *HACD1* have progressively accentuated clinical features that generally stabilize (4,10). The mutant dogs suffered from congenital myopathy with severe hypotonia. Histologic analysis of this dog model reveals CNM along with type 1 fiber atrophy and a type 2 fiber deficiency in skeletal muscle, which mimics human autosomal dominant and recessive forms of CNM (10). In contrast to dogs, the histology studies in our patients could not establish the centronuclear form of the myopathy. This may be attributed to the young age at which histological analyses were performed in our patients, and is in correlation with the age effect noted in dogs in which centrally located nuclei were observed in only 30% of myofibers at age 3, while at the age of 7 years, centralization was more obviously noted and detected in as much as 60% of fibers (10). Alternatively, the differences in myopathies in humans versus dogs could result from the difference in modifier genes contributing to the variable clinical stigmata. This is exemplified by the identification of a mutation in *NPHP4* leading to cone-rod dystrophy a retinal degenerative disease in dogs (11), whereas in humans mutations in this gene are reported to cause both cone-rod dystrophy and nephronophthisis leading to end stage renal failure. Finally, the *HACD1* mutation in humans as detected in our patients differs from that identified in dogs leading directly to the variability in clinical stigmata between species.

VLCFAs are fatty acids with a chain-length of >20 carbons that function in numerous cellular processes, including sphingolipid biogenesis, skin barrier formation, sperm development and maturation, fetal growth and development, retinal functions, brain development, inflammation and maintenance of myelin (12). VLCFA elongation takes place by cycling through a four-step process (condensation, reduction, dehydration and reduction), with two carbons added through each cycle. HACD1/PTPLA and three paralogs (HACD2/PTPLB, HACD3/PTPLAd1 and HACD4/PTPLAd2) are responsible for the third step, dehydration, converting 3-hydroxyacyl-CoA to 2,3-*trans*-enoyl-CoA (6). *HACDs* are differently expressed among the variable human tissues: *HACD1* is mainly expressed in skeletal muscles and heart (13) in agreement with our finding that a mutation in *HACD1* affects muscle; however, cardiac activity was spared in our patients. *HACD2* and *HACD3* are ubiquitously expressed, but with lower levels of *HACD3* in skeletal muscle (6,13). In contrast, *HACD4* is highly expressed in leukocytes only, with diminished expression in heart, spleen, kidney, placenta and lung (6). In line with the known tissue specific expression control, it is possible that the relatively mild effect observed in our patients despite the abrogation of VLCFA synthesis by *HACD1* is the result of compensating activities by *HACD2* and *HACD3*. It could be further speculated that the improvement with age detected in our patients is the result of temporal regulation resulting in *HACD2* or *HACD3* taking over the role of *HACD1* as patients mature. It is also possible that interplay with other modifiers decrease the clinical findings with age. Although HCAD1 is highly expressed in human heart (13), no major early-onset specific cardiac dysfunction was observed either in our patients or in the dog model. This is consistent with the recent exclusion of *HACD1* as an arrhythmogenic factor (14). Although HCAD1 has been shown to be expressed in some parts of the brain (13), its impairment does not affect cognitive functions as these along with brain imaging appear to be normal in our patients.

Protein phosphorylation of tyrosine residues plays a pivotal role in development and physiology by regulating cell proliferation, differentiation, growth, migration and motility (15). *HACD1* (also known as *PTPLA*) belongs to the family of PTPL proteins that have a conserved PTPase catalytic motif, except for the substitution of a catalytically active proline for arginine making it possible that HACD1 may not be a tyrosine kinase (16). *HACD1* was demonstrated to be required for myoblast growth and differentiation. Furthermore, it was identified as a target gene of the serum response factor in skeletal- and cardiac-muscle-specific cells (17). HACD1 activity is expected to be drastically reduced in our patients because of non-sense mediated decay, and in agreement with the myopathic presentation of our patients. The HACD protein is localized to the ER membrane (6). We previously examined the membrane topology of the yeast HACD1 homolog Phs1 and determined it to be a six-span membrane protein with its N- and C-terminus both facing toward the cytosol (18). Residues important or essential for the enzyme activity of Phs1 are located within and around the transmembrane regions 3 and 5 (18). The non-sense mutation in residue 248 causes the elimination of the transmembrane region 6. Although the predicted mutant protein still contains all these important residues, truncation of the entire transmembrane region 6 would likely disrupt structural integrity of the HACD1 protein. In support of this, it should be noted that the mutant protein was unexpectedly *N*-glycosylated. Since HACD1 protein contains only one *N*-glycosylation motif (NXS or NXT) at Asn243-Val244-Ser245, glycosylation must occur at the Asn243 residue. Considering the membrane topology of Phs1, Asn243 in wild-type HACD1 should be located in the third ER luminal loop. However, this loop is converted to the C-terminal tail by the Y248Stop mutation. We speculate that the loop-to-tail conversion causes structural changes in the Asn243 region or increases the distance between the ER membrane and Asn243, leading to accessibility of Asn243 to oligosaccharyltransferase resulting in glycosylation.

Since the HACD1 protein catalyzes an essential step in VLCFA synthesis, VLCFA levels and lipid composition may be affected in the skeletal muscle of patients with the *HACD1* (*Y248Stop*) mutation. Involvement of VLCFAs in vesicular trafficking and maintenance of organelle structure/functions has been reported in yeast (19–23). VLCFAs are long enough to span both leaflets of the lipid bilayer, which would facilitate stabilization of highly curved membranes. Such membrane-stabilizing functions of VLCFAs have been proposed for the maintenance of the functional nuclear pore complex, around which highly curved membranes like T-tubules, a functionally important structure of muscle, exist (19). Lipids play active roles in the organization, shaping and function of membranes (24). Besides their structural function, lipids are also linked to important physiological processes such as bioenergetics, cell recognition, signal transduction and apoptosis (25,26). This may explain the finding that HACD1 is required for myoblast growth and differentiation and serves as a target gene for the serum response factor in skeletal muscle-specific cells.

In summary, we demonstrate that impaired HACD1 enzyme activity, involved in third step elongation of VLCFA, causes congenital myopathy that improves with age. This novel implication of VLCFA in normal muscle cell activity contributes to the understanding of the disease mechanism and possible future treatment, as well as to diagnosis and family planning.

## MATERIALS AND METHODS

The study was approved by the institutional review board, and all the participants gave written informed consent prior to participation. Genomic DNA was extracted from white blood cells by using standard procedures.

### Genotyping and exome sequencing

Genotyping was performed using Affymetrix (CA, USA) GeneChip Human SNP5 array. We determined the genotype calls by using Affymetrix GeneChip Genotyping Console Analysis Software. KinSNP (27) software was used to automatically search the microarray results for homozygous regions consistent with linkage.

Whole-exome sequencing was performed using NimbleGen v2 exome capture followed by 50 bp paired end sequencing using an Illumina HiSeq Sequencing System. The resulting sequence data were analyzed using the recommended best practices from the 1000 Genomes project. Briefly, this process included mapping to the human reference genome using BWA (28) and removing duplicate reads with Picard tools (http://picard.sourceforge.net). Realignment around insertions and deletions, quality score recalibration and variation calling was done using GATK (29,30). Variations were annotated using a custom annotation program developed by the University of Iowa.

### Verification of a mutation

To verify the mutation of genomic DNA, PCR amplification of exon 6 was performed using primers F: 5′-TGTAAAGAAAAGAGAATTTTGAAGTTT-3′ and R: 5′-TCCCTTCACTTTTCCCAAAT-3′ (PCR conditions were 94°C for 3 min followed by 40 cycles of 94°C for 40 s, 59°C for 40 s and 72°C for 1 min, with final extension step of 7 min at 72°C). Direct sequencing the PCR products was performed on an ABI PRISM 3100 DNA Analyzer with the BigDye Terminator v.1.1 Cycle Sequencing Kit (Applied Biosystems, CA, USA) according to the manufacturer's protocol. The change creates an SspI restriction site. All patients and available parents were verified for the change by SspI restriction. The PCR generated a 455-bp amplicon that is cleaved by the enzyme into 195 and 260 bp fragments, separated by electrophoresis on 2% agarose gel.

### Quantitative RT–PCR to verify mRNA decay

To test this hypothesis, we extracted RNA from the Tek-OCT (Sarkura) embedded frozen skeletal muscle biopsy of Patient III-5 using the EZ-RNA II Kit (Biological Industries, Israel). For the control sample, we used human skeletal muscle total RNA (Ambion). Reverse transcriptase reactions were performed with 1μg RNA from patient and control using the Super-Script II Reverse Transcriptase Synthesis Kit (Invitrogen) with hexamers random primers. For qPCR on ABI 7500 Real-Time PCR System (Applied Biosystems), we mixed cDNA, SYBR Green (Applied Biosystems) and the *HACD1* primers: F: 5′-CGCATGTGAAGAAAACAGGA-3′, R: 5′-CGTAACATATGAAAATAGAGTTGTGGA-3′ (exons 5 and 6, respectively) or *GAPDH* primers F: 5′-AGAAGGCTGGGGCTCATTTG-3′, R: 5′-AGGGGCCATCCACAGTCTTC-3′ (Shanghai ShineGene Molecular Biotech). PCR conditions were 2 min 50°, and 95° 10 min followed by 40 cycles at 95° for 15 s and 60° 1 min. The analyzed qPCR data were analyzed with the ABI 7500 Software V2.0.3 (Δ*C*_t_ method; normalization against GAPDH). The *HACD1* primers yielded a linear standard curve with an *R*^2^ = 0.99. The melting curve proved amplification of one product.

### Construction of the HACD1 mutation

To verify the effect of the truncation on the known enzymatic activity of HACD1/ PTPLA, the non-sense mutation was introduced into pCE-puro 3xFLAG-HACD1 plasmid (6) by site directed mutagenesis using the Phusion Hot Start II high-fidelity DNA polymerase (Finnzymes) and the primers F: 5′-GTCTCTTTTGACTA**A**TATTATTTTCTTC-3′ and R: 5′-GAAGAAAATAATA**T**TAGTCAAAAGAGAC-3′.

### Biochemical assays

HEK 293 T cells were transfected with the wild-type or mutated constructs, and the FLAG-tagged proteins were affinity-purified using anti-FLAG M2 agarose (Sigma) as described previously (18).

The CoA dehydratase *in vitro* assay was performed as described previously (14) using 0.01 μCi [^14^C]3-hydroxypalmitoyl-CoA (55 mCi/mmol; American Radiolabeled Chemicals, St. Louis, MO, USA) as substrate. Immunoblotting was performed as described previously (14) using anti-FLAG antibody M2 (1 μg/ml; Stratagene, Agilent Technologies, La Jolla, CA, USA) as the primary antibody. Deglycosylation of proteins was performed as described previously (31) using PNG F or Endo H (New England Biolabs, Beverly, MA, USA).

## SUPPLEMENTARY MATERIAL

Supplementary Material is available at *HMG* online.

*Conflict of Interest statement*. None declared.

## FUNDING

Funding to pay the Open Access publication charges for this article was provided by the Howard Hughes Medical Institute.

## Supplementary Material

Supplementary Data

## References

[1] Nance J.R., Dowling J.J., Gibbs E.M., Bönnemann C.G. (2011). Congenital myopathies: an update. Curr. Neurol. Neurosci. Rep..

[2] Kaplan J.C. (2011). The 2012 version of the gene table of monogenic neuromuscular disorders. Neuromuscul. Disord..

[3] Iannaccone S.T., Bove K.E., Vogler C., Azzarelli B., Muller J. (1986). Muscle maturation delay in infantile myotonic dystrophy. Arch. Pathol. Lab. Med..

[4] Pelé M., Tiret L., Kessler J.L., Blot S., Panthier J.J. (2005). SINE Exonic insertion in the PTPLA gene leads to multiple splicing defects and segregates with the autosomal recessive centronuclear myopathy in dogs. Hum. Mol. Genet..

[5] Maquat L.E. (2004). Nonsense-mediated mRNA decay: splicing, translation and mRNP dynamics. Nat. Rev. Mol. Cell Biol..

[6] Ikeda M., Kanao Y., Yamanaka M., Sakuraba H., Mizutani Y., Igarashi Y., Kihara A. (2008). Characterization of four mammalian 3-hydroxyacyl-CoA dehydratases involved in very long-chain fatty acid synthesis. FEBS Lett..

[7] Buj-Bello A., Biancalana V., Moutou C., Laporte J., Mandel J.L. (1999). Identification of novel mutations in the MTM1 gene causing severe and mild forms of X-linked myotubular myopathy. Hum. Mutat..

[8] Lev D., Sadeh M., Watemberg N., Dabby R., Vinkler C., Ginzberg M., Lerman-Sagie T. (2006). A benign congenital myopathy in an inbred Samaritan family. Eur. J. Paediatr. Neurol..

[9] Böhm J., Leshinsky-Silver E., Vassilopoulos S., Le Gras S., Lerman-Sagie T., Ginzberg M., Jost B., Lev D., Laporte J. (2012). Samaritan myopathy, an ultimately benign congenital myopathy, is caused by a RYR1 mutation. Acta. Neuropathol..

[10] Tiret L., Blot S., Kessler J.L., Gaillot H., Breen M., Panthier J.J. (2003). The cnm locus, a canine homologue of human autosomal forms of centronuclear myopathy, maps to chromosome 2. Hum. Genet..

[11] Wiik A.C., Wade C., Biagi T., Ropstad E.O., Bjerkås E., Lindblad-Toh K., Lingaas F. (2008). A deletion in nephronophthisis 4 (NPHP4) is associated with recessive cone-rod dystrophy in standard wire-haired dachshund. Genome Res..

[12] Kihara A. (2012). Very long-chain fatty acids: elongation, physiology, and related disorders. J. Biochem..

[13] Li D., Gonzalez O., Bachinski L.L., Roberts R. (2000). Human protein tyrosine phosphatase-like gene: expression profile, genomic structure, and mutation analysis in families with ARVD. Gene.

[14] Konishi H., Okuda A., Ohno Y., Kihara A. (2010). Characterization of HACD1 K64Q mutant found in arrhythmogenic right ventricular dysplasia patients. J. Biochem..

[15] Li L., Dixon J.E. (2000). Form, function, regulation of protein tyrosine phosphatases and their involvement in human diseases. Immunology.

[16] Wang B., Pelletier J., Massaad M.J., Herscovics A., Shore G.C. (2004). The yeast split-ubiquitin membrane protein two-hybrid screen identifies BAP31 as a regulator of the turnover of endoplasmic reticulum-associated protein tyrosine phosphatase-like B. Mol. Cell. Biol..

[17] Lin X., Yang X., Li Q., Ma Y., Cui S., He D., Lin X., Schwartz R.J., Chang J. (2012). Protein tyrosine phosphatase-like A regulates myoblast proliferation and differentiation through MyoG and the cell cycling signaling pathway. Mol. Cell. Biol..

[18] Kihara A., Sakuraba H., Ikeda M., Denpoh A., Igarashi Y. (2008). Membrane topology and essential amino acid residues of Phs1, a 3-hydroxyacyl-CoA dehydratase involved in very long-chain fatty acid elongation. J. Biol. Chem..

[19] Schneiter R., Hitomi M., Ivessa A.S., Fasch E.V., Kohlwein S.D., Tartakoff A.M. (1996). A yeast acetyl coenzyme A carboxylase mutant links very-long-chain fatty acid synthesis to the structure and function of the nuclear membrane-pore complex. Mol. Cell. Biol..

[20] David D., Sundarababu S., Gerst J.E. (1998). Involvement of long chain fatty acid elongation in the trafficking of secretory vesicles in yeast. J. Cell. Biol..

[21] Chung J.H., Lester R.L., Dickson R.C. (2003). Sphingolipid requirement for generation of a functional V_1_ component of the vacuolar ATPase. J. Biol. Chem..

[22] Gaigg B., Timischl B., Corbino L., Schneiter R. (2005). Synthesis of sphingolipids with very long chain fatty acids but not ergosterol is required for routing of newly synthesized plasma membrane ATPase to the cell surface of yeast. J. Biol. Chem..

[23] Yu L., Pena Castillo L., Mnaimneh S., Hughes T.R., Brown G.W. (2006). A survey of essential gene function in the yeast cell division cycle. Mol. Biol. Cell.

[24] Kornmann B., Roux A. (2012). Lipids as organizers of cell membranes. EMBO Rep..

[25] Engelmann B., Wiedmann M.K.H. (2010). Cellular phospholipid uptake: flexible paths to coregulate the functions of intracellular lipids. Biochim. Biophys. Acta.

[26] Osman C., Voelker D.R., Langer T. (2011). Making heads or tails of phospholipids in mitochondria. J. Cell. Biol..

[27] El-ad A., Bartal O., Morad E., Nagar T., Sheynin J., Parvari R., Chalifa-Caspi V. (2010). KinSNP software for homozygosity mapping of disease genes using SNP microarrays. Hum. Genomics.

[28] Li H., Durbin R. (2009). Fast and accurate short read alignment with Burrows-Wheeler *transform*. Bioinformatics.

[29] McKenna A., Hanna M., Banks E., Sivachenko A., Cibulskis K., Kernytsky A., Garimella K., Altshuler D., Gabriel S., Daly M. (2010). The Genome Analysis Toolkit: a MapReduce framework for analyzing next-generation DNA sequencing data. Genome Res..

[30] DePristo M., Banks E., Poplin R., Garimella K., Maguire J., Hartl C., Philippakis A., del Angel G., Rivas M.A., Hanna M., McKenna A. (2011). A framework for variation discovery and genotyping using next-generation DNA sequencing data. Nat. Genet..

[31] Ohno Y., Ito A., Ogata R., Hiraga Y., Igarashi Y., Kihara A. (2009). Palmitoylation of the sphingosine 1-phosphate receptor S1P1 is involved in its signaling functions and internalization. Genes Cells.

