# Supplementary material for: Congenital myopathy is caused by mutation of HACD1
Source: Hum Mol Genet. 2013 Aug 9;22(25):5229–36. doi: 10.1093/hmg/ddt380 (PMC3842179; doi:10.1093/hmg/ddt380)
Supplement: Supplementary Data [file supp_22_25_5229__index.html]

Congenital Myopathy is Caused by Mutation of HACD1 — Congenital myopathy is caused by mutation of HACD1 — Congenital myopathy is caused by mutation of HACD1 — Supplementary Data 

# Congenital myopathy is caused by mutation of *HACD1*

## Supplementary Data

Supplementary Data

**Files in this Data Supplement:**

- Supplementary Data - Doc file
- Supplementary Figure 1 - tif file
- Supplementary Figure 2 - tif file
