# Supplementary material for: Congenital myopathy is caused by mutation of HACD1
Source: Hum Mol Genet. 2013 Aug 9;22(25):5229–36. doi: 10.1093/hmg/ddt380 (PMC3842179; doi:10.1093/hmg/ddt380)
Supplement: Supplementary Data [file supp_ddt380_ddt380supp.doc]

**Supplementary figures:**

**Figure 1: Immunohistochemical analyses of the open biopsy of Patient III-5.**

Representative panel of immunohistochemical stains that were performed on the biopsy of patient III-5. There is normal sarcolemmal staining for dystrophin (A [dys2]), sarcoglycans (B [alpha-sarcoglycan]), caveolin-3 (C), laminin beta-1 (D), merosin (E [80KD], F [300KD]), collagen IV (G), collagen VI (H), and normal nuclear staining for emerin (I).

**Figure 2: Electron microscopy analysis of the core needle biopsy of patient III-8.**

Electron microscopy was performed on the biopsy of patient III-8. No diagnostic abnormalities were detected. No internal nuclei were seen. The myofibrillar structure, including all bands and lines appeared to be normal. Scattered small lipid droplets (black arrows), consistent with type 1 myofiber are noted. Subsarcolemmal accumulation of unremarkable mitochondria (white arrows) is seen. Circular profiles comprising normal triads and adjacent mitochondria (dashed arrow) are evident in between myofibrils.
