# Supplementary figures and images for: Congenital myopathy is caused by mutation of HACD1
Source: Hum Mol Genet. 2013 Aug 9;22(25):5229–36. doi: 10.1093/hmg/ddt380 (PMC3842179; doi:10.1093/hmg/ddt380)

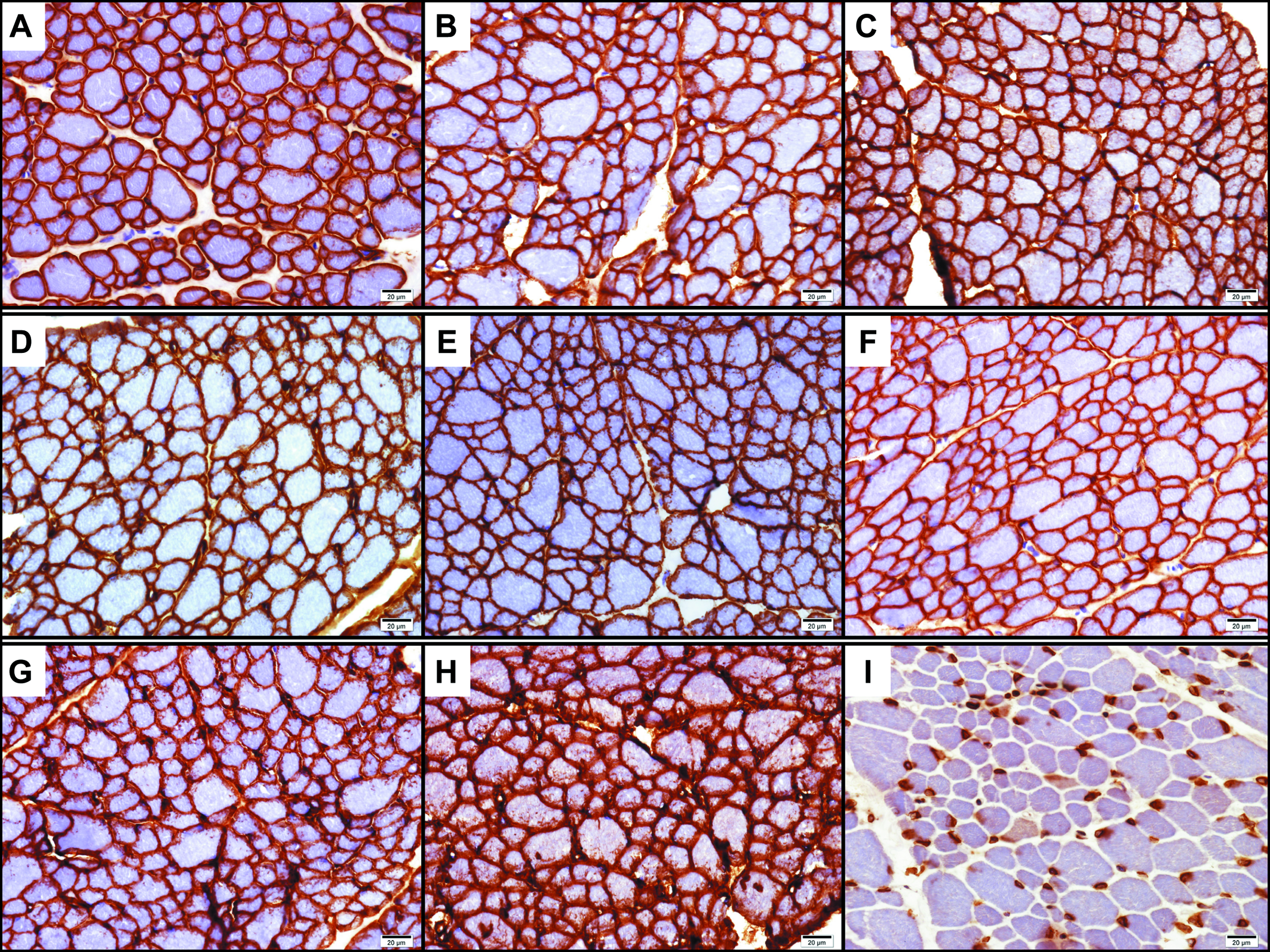

Supplement: Supplementary Data [file supp_ddt380_ddt380supp_fig1.tif]

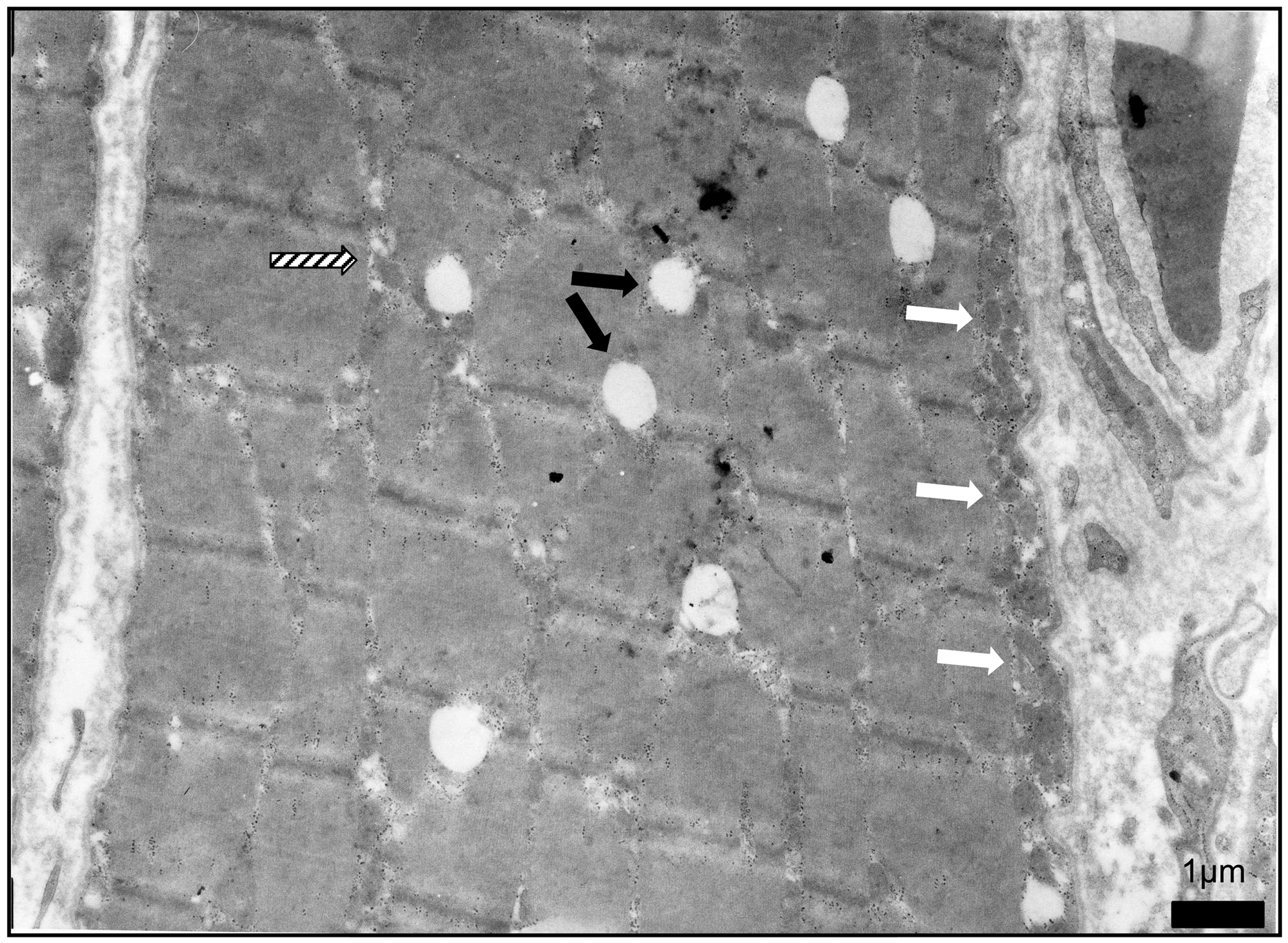

Supplement: Supplementary Data [file supp_ddt380_ddt380supp_fig2.tif]
